# RETRACTED: Muniba; Yu, B. Does Innovative City Pilot Policy Stimulate the Chinese Regional Innovation: An Application of DID Model. *Int. J. Environ. Res. Public Health* 2023, *20*, 1245

**DOI:** 10.3390/ijerph22071127

**Published:** 2025-07-17

**Authors:** Muniba, Baorong Yu

**Affiliations:** School of Insurance and Economics, University of International Business and Economics, Beijing 100029, China

The journal retracts the article “Does Innovative City Pilot Policy Stimulate the Chinese Regional Innovation: An Application of DID Model” [1], cited above.

Following publication, concerns were brought to the attention of the Editorial Office regarding an overlap between this publication [1] and a published manuscript [2] produced by a different author group.

Adhering to our complaints procedure, the Editorial Office and Editorial Board conducted an investigation that confirmed a significant overlap between these two articles [1,2], without appropriate acknowledgment. As a result, the Editorial Board have decided to retract this paper as per MDPI’s retraction policy (https://www.mdpi.com/ethics#_bookmark30).

This retraction was approved by the Editor-in-Chief of the *International Journal of Environmental Research and Public Health*.

Baorong Yu agrees to this retraction. Muniba disagrees to this retraction.
